# Aneurysm of Brachial Artery Following Axillary Crutch

**Published:** 2011-04-01

**Authors:** J Vahedian-Ardakani, M Vahedian, F Nabavizadeh

**Affiliations:** 1 Department of Surgery, Firuzgar Hospital, Tehran University of Medical Sciences, Tehran, Iran; 2 Department of Orthopedic Surgery, Emam-Khomeini Hospital, Tehran University of Medical Sciences, Tehran, Iran; 3 Department of Physiology, Tehran University of Medical Sciences, Tehran, Iran

**Keywords:** Aneurysm, Brachial Artery, Crutch

Dear Editor,

True aneurysm of brachial artery, just distal to axillary artery, is relatively rare. Moreover, this type of aneurysm following usage of axillary crutch has not reported in the literature. Furthermore, this entity presenting as Reynaud’s disease has not been described; though microemboli following subclavio-axillary artery aneurysm (arterial thoracic outlet syndrome) is well-defined. We report a case of large fusiform aneurysm of the brachial artery, following long time usage of unilateral axillary crutch, complicated by Reynaud’s phenomenon and complete outflow obstruction that presented as acute limb ischemia.

**Fig. 1:**
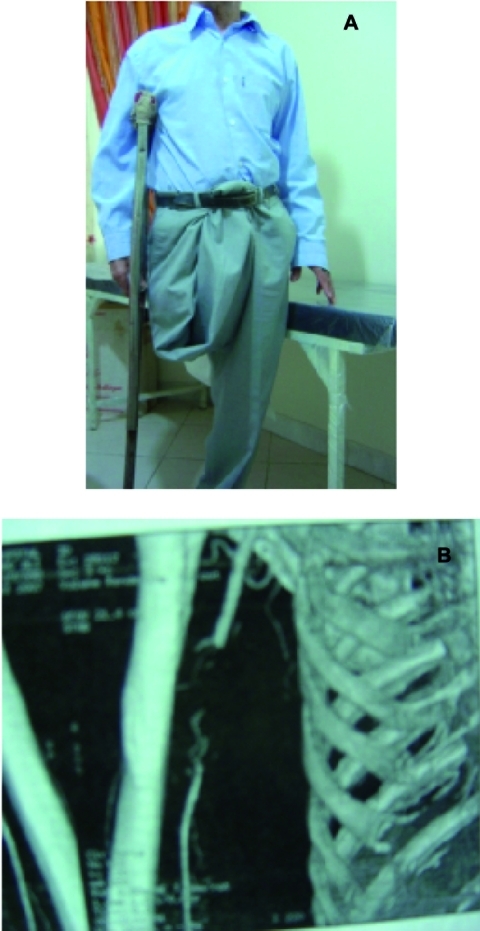
A: The patient spent 50 years on unilateral Crutch, B: CT angiogram showed cut-off in proximal part of brachial artery, due to thrombosed aneurysm, and run-off in distal

A 64-year-old man was referred to the emergency unit, with symptoms and signs of acute limb ischemia of right upper extremity. The patient recounted that he had elective right cervical sympathectomy because of painful and non-healing ulcer of the tip of index finger. Following repeated coldness and painful events of his hand, all affected fingers of the hand became atrophic with decreased range of motion. On examination, the hand was cold and painful, without palpable pulses distal to the axillary artery. There was a palpable fusiform mass about 5x3 centimeter compatible with brachial artery, just distal to the axillary artery. The other peripheral pulses were normal. The right fingers showed signs of acute ischemia. His comorbidity included above-the-knee amputation of ipsilateral lower extremity in childhood following traffic accident. After that, he spent 50 years on unilateral crutch (Figure 1A). He was a cigarette smoker one pack/day for 45 years. A duplex scan demonstrated a normal triphasic flow in axillary artery, but biphasic pattern in distal brachial and forearm arteries. CT angiography revealed a cut-off in proximal part of the brachial artery and run-off the distal part (Figure 1B). The patient underwent emergency exploration of the right brachial artery. There was a fusiform aneurysm just distal to the axillary artery that obstructed it completely. The affected segment was removed and replaced by a saphenous vein graft.[1][2] The affected hand became warm similar to contralateral hand, but digital pulses did not recover, though wrist pulses became palpable but diminished in comparison to the other side. Histology of the specimen confirmed a fibrous-walled saccular true aneurysm 2.5 centimeter diameter, containing organized and fresh thromboses. All three layers of the aneurysmal artery were intact, with atheromatous changes of intimo-medial layer.

Upper extremity aneurysms are uncommon, and are most commonly false aneurysms.[1] True aneurysm of the brachial and more distal arteries are rare.[2] Of 581 procedures involving brachial artery performed at the Cleveland Clinic Foundation between January 1989 and December 2000, only three were repairs of brachial artery aneurysms. Two were iatrogenic false aneurysms following cardiac intervention; and only one was a true aneurysm of degenerative origin (1/581; 0.172%), as supported by arteriomegaly of the entire brachial artery on arteriography and by the histological appearance of the operative specimen.[3] Based on our knowledge, true brachial artery aneurysm just distal to the axillary artery following long term usage of a crutch has not been described. Moreover, presentation as Reynaud’s phenomenon and Stellate ganglion sympathectomy has not been reported.

In over 80 percent of patients with Reynaud’s phenomenon who are seen by an internist, the condition is simply an exaggeration of the physiologic response to cold temperatures. However, as in this case, it can represent a clinical manifestation of a serious underlying disease or be the first sign of critical ischemia of a digit or limb. Depending on the severity of the vascular insult and the size of the vessel involved, superficial ulceration or deep-tissue necrosis with gangrene and amputation can result. Clinical criteria are used to distinguish patients with uncomplicated, or primary, Reynaud’s phenomenon from those with secondary Reynaud’s phenomenon.[4][5]

The suggested criteria for primary Reynaud’s phenomenon are symmetric attacks; the absence of tissue necrosis, ulceration, or gangrene; the absence of a secondary cause on the basis of a patient’s history and general physical examination; normal nail fold capillaries; a negative test for antinuclear antibody; and a normal erythrocyte sedimentation rate.[5] The median age at the onset of primary Reynaud’s phenomenon is 14 years. A secondary cause of Reynaud’s phenomenon is suggested by the following findings: age at onset of more than 30 years; episodes that are intense, painful, asymmetric, or associated with ischemic skin lesions; clinical features suggestive of a connective-tissue disease (e.g., arthritis and abnormal lung function); specific autoantibodies; and evidence of microvascular disease on microscopy of nail-fold capillaries.[6][7] This case had asymmetric, intensely painful attacks of affected limb, history of finger ulcer, and without any evidence of connective tissue disorder. It is unclear why this obviously secondary Reynaud’s phenomenon has not been distinguished. Report of this case put emphasis on the importance of meticulous history taking and physical examination for proper diagnosis and selecting appropriate therapy.

All patients with Reynaud’s phenomenon need to undergo a complete evaluation to rule out an underlying secondary cause, with specialized tests performed as appropriate on the basis of the clinical history. Patients who have a history of single digit or asymmetric attacks, absent pulses, asymmetry of blood pressure, or evidence of critical ischemia should undergo arterial Doppler ultrasonography or angiography. Long term crutch may complicated by axillobrachial artery injury due to repeated minor physical trauma. The role of proximal sympathectomy in the management of Reynaud’s phenomenon has not been established.
